# Differentiating Wheat Genotypes by Bayesian Hierarchical Nonlinear Mixed Modeling of Wheat Root Density

**DOI:** 10.3389/fpls.2017.00282

**Published:** 2017-03-02

**Authors:** Anton P. Wasson, Grace S. Chiu, Alexander B. Zwart, Timothy R. Binns

**Affiliations:** ^1^Commonwealth Scientific and Industrial Research Organisation (CSIRO) Agriculture & FoodCanberra, ACT, Australia; ^2^Research School of Finance, Actuarial Studies and Statistics, College of Business and Economics, Australian National UniversityCanberra, ACT, Australia; ^3^Commonwealth Scientific and Industrial Research Organisation (CSIRO) Data61Canberra, ACT, Australia; ^4^Australian Taxation OfficeSydney, NSW, Australia

**Keywords:** generalized linear mixed models, heritability, hierarchical modeling, root architecture, wheat phenotyping

## Abstract

Ensuring future food security for a growing population while climate change and urban sprawl put pressure on agricultural land will require sustainable intensification of current farming practices. For the crop breeder this means producing higher crop yields with less resources due to greater environmental stresses. While easy gains in crop yield have been made mostly “above ground,” little progress has been made “below ground”; and yet it is these root system traits that can improve productivity and resistance to drought stress. Wheat pre-breeders use soil coring and core-break counts to phenotype root architecture traits, with data collected on rooting density for hundreds of genotypes in small increments of depth. The measured densities are both large datasets and highly variable even within the same genotype, hence, any rigorous, comprehensive statistical analysis of such complex field data would be technically challenging. Traditionally, most attributes of the field data are therefore discarded in favor of simple numerical summary descriptors which retain much of the high variability exhibited by the raw data. This poses practical challenges: although plant scientists have established that root traits do drive resource capture in crops, traits that are more randomly (rather than genetically) determined are difficult to breed for. In this paper we develop a hierarchical nonlinear mixed modeling approach that utilizes the complete field data for wheat genotypes to fit, under the Bayesian paradigm, an “idealized” relative intensity function for the root distribution over depth. Our approach was used to determine *heritability*: how much of the variation between field samples was purely random vs. being mechanistically driven by the plant genetics? Based on the genotypic intensity functions, the overall heritability estimate was 0.62 (95% Bayesian confidence interval was 0.52 to 0.71). Despite root count profiles that were statistically very noisy, our approach led to denoised profiles which exhibited rigorously discernible phenotypic traits. Profile-specific traits could be representative of a genotype, and thus, used as a quantitative tool to associate phenotypic traits with specific genotypes. This would allow breeders to select for whole root system distributions appropriate for sustainable intensification, and inform policy for mitigating crop yield risk and food insecurity.

## 1. Introduction

Meeting the food production requirements of a growing human population who are encroaching on arable land and generating a changing climate will require an intensification of agriculture, where greater yields are obtained from crops on existing farms with sustainable inputs of water and fertilizer (Gregory et al., 2013). This will involve identifying the constraints on yield in agricultural systems, many of which are to be found below ground in the root systems of crops. There are calls for a “second Green Revolution” (Lynch, 2007) focused on breeding crops with “ideotypic” (Donald, 1968) root systems (i.e., possessing desirable root system traits) that can overcome these constraints. This approach, called physiological breeding, is to be contrasted with breeding for increased yield alone, an approach which is no longer keeping pace with growing demands (Fischer and Edmeades, 2010; Richards et al., 2010; Hall and Richards, 2013).

However, identifying ideotypic root systems for crops is fraught with difficulty. Root traits which can be identified in the laboratory are often difficult to translate to the field (Watt et al., 2013) because they are devoid of the developmental context of the soil. The soil environment is complex, and has a dominant effect on root system development (Rich and Watt, 2013). Furthermore, crop physiological models—which are used to formulate strategies for plant breeding and crop yield risk mitigation, and even to develop government policy—are often inadequate in addressing the spatial heterogeneity of root systems and soil properties (Holzworth et al., 2014). It is also difficult to sample roots in soil in the field, and the data obtained are complex to interpret. Nevertheless, it is in the field where the effects of soil, climate, and agronomy are integrated with the developmental genetics of the plants growing together as a crop, and hence it is also in the field where measuring root traits, identifying crop ideotypes, and modeling root development are most valuable. Selecting for root ideotypes in the field may speed up the identification of the best germplasm for breeding programs (Wasson et al., 2012; Rich and Watt, 2013).

Therefore, integrating improved measures of root distribution/development into crop physiological models will improve farm management decision making and crop yield risk mitigation. Yet, indirect measurements of crop root systems are problematic, and most direct measurements are destructive, time-consuming and/or labor intensive (e.g., root washing, minirhizotrons) (Wasson et al., 2012). Hence the core-break method was developed as a method of rapidly observing and quantifying the presence of roots as a function of depth (Drew and Saker, 1980; van Noordwijk et al., 2001); a soil core sample is taken from the crop and broken at regular intervals (corresponding to increasing depth) and the exposed roots are counted. The counts correlate with the root length in the corresponding volume of soil. This technique has been used to phenotype root count distributions in 43 genotypes (Wasson et al., 2014) and efforts have been made to automate the root count process (Wasson et al., 2016) to reduce the labor requirements. However, root counts from the core-break method are subject to a high degree of variation between samples (van Noordwijk et al., 2001), which makes it challenging to identify genotype-specific traits from root counts or to associate genotypes with discernible properties of root count profiles.

Similar types of experimental field data may have been analyzed by statistical linear models (Faraway, 2014) under an analysis-of-variance (ANOVA) framework (Wasson et al., 2014). However, a major limitation of linear models is their assumption of Gaussian (normally distributed) response data, whereas root counts are discrete, bounded below by zero, and with a distribution whose substantial right-skewness may not be easily removed by variable transformation. Indeed, root count data are more appropriately modeled as Poisson distributed, although a phenomenon known as *overdispersion* (McCulloch, 2008), commonly encountered in count data from field experiments, must be handled with care. More specifically, the Poisson distribution is characterized by a single parameter that represents the distribution mean as well as its variance. However, in practice, the count variable of concern often has a recognizably larger variance than its mean (hence, “overdispersion”), although the overall distribution still resembles Poisson in other respects.

Therefore, linear models applied to field data thus far have focused on analyzing core-level summary metrics, such as maximum rooting depth, which, after variable transformation if necessary, can approximately behave as Gaussian (Wasson et al., 2014). However, such summary metrics by definition cannot reflect root structure over depth, discarding valuable information contained at the level of individual core segments, and consequently resulting in an undesirable loss of statistical power.

**Figure 1:**
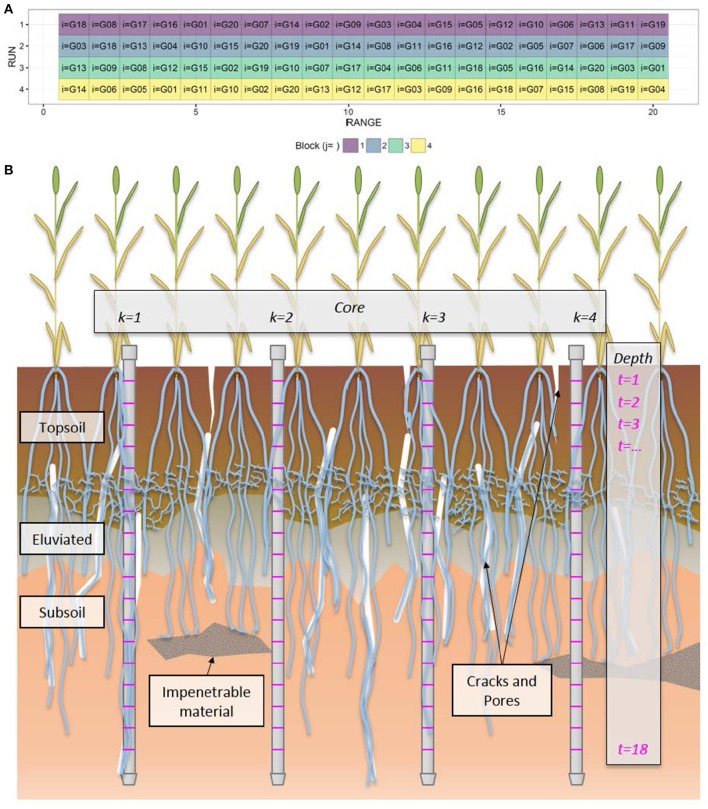
**Schematics of the field experiment. (A)** Surface layout of the field experiment involving twenty genotypes (indexed by *i*) randomized within four blocks (indexed by *j*) of twenty ranges of plots. **(B)** Cartoon depicting the sampling in each plot. Four soil cores (indexed by *k*) were sampled from each plot in a steel tube. Each core was broken into 10 cm increments. The root count (*y*) at each 10 cm depth increment (indexed by *t*) is the sum of the counts on the lower face of the upper fragment and the upper face of the lower fragment. Thus, each root count, *y*_*ijkt*_, has four unique index values. The cartoon further depicts the variability that might be encountered by sampling soil cores in a single plot, e.g., contrast cores *k* = 1 and *k* = 4.

To better facilitate our scientific objective of associating genotypes with discernible properties of root count profiles, in this paper we scrutinize the many facets of the inherent variability of the root count profile produced based on a field trial (Figure 1) that involved twenty genotypes (*n*_*G*_ = 20), each generating four replicated soil cores (*n*_*C*_ = 4) extracted *in situ* from each of four replicated plots or blocks (*n*_*B*_ = 4). Growing in a plot, as they would in a farmer's field, the plants' root systems interact and respond to each other. Their development is driven by the exploration of cracks and pores (White and Kirkegaard, 2010), which are randomly distributed. Likewise, variation in soil chemistry and nutrients, which can be patchy and vary with depth, drives the branching of roots. In contrast, impenetrable material and compaction can inhibit growth. As each soil core only captures a comparatively small piece of variation due to the various sources, results found in adjacent replicated cores can differ substantially.

Moreover, we note that many of the profiles of the average root count depicted in Figure 6 of Wasson et al. (2014) are consistent with random observations whose mean follows a functional form that roughly resembles the density function of the gamma probability distribution. Based on this observation, in this paper we develop a statistical modeling approach that can rigorously handle the non-standard nature of our root count data. Specifically, root counts at the observed depths (denoted by *t*) within a core are formally related through a nonlinear parametric expression θ(*t*) to reflect the one-dimensional spatial nature of individual soil cores. The parametric expression (with a small number of unknown parameters) is the common denominator that unifies this spatial behavior among all cores. Obviously, an appropriate parametric structure imposed on the root counts within a core would lead to much greater statistical power when compared to, say, an oversimplified ANOVA approach that regards depth as a mere design feature in a factorial experiment.

We also note that our field experimental setup was such that the randomness in our data exhibits a hierarchical structure (Gelman and Hill, 2006) that comprises layers of mean and variance functions. In particular, the complex, non-standard experimental design features inherent in our data require hierarchical nonlinear mixed modeling (HNLMM), an approach which addresses our need to model overdispersed, Poisson distributed data (McCulloch, 2008) via a hierarchy of nonlinear mean functions and associated variance components due to the formulation of θ(*t*).

Therefore, our approach in this paper is distinguished from existing studies particularly because of (a) our scrutiny of the root count profiles themselves, rather than the relationship between the counts and the root length density, and (b) our hierarchical modeling approach that integrates all identified facets of variability among all observed root count profiles in a comprehensive and collective manner. Additionally, our modeling framework gives rise to new heritability metrics that describe spatial and overall root architectural traits, the latter at the overall genotypic level.

The remainder of our paper is structured as follows. Under the section Materials and Methods, we provide some details of the field sampling procedure that gave rise to our root count profile data, some data visualizations, a primer on the specification of our HNLMM under a Bayesian framework (Gelman et al., 2013), and biological interpretations of model parameters and their use in defining novel multiresolution heritability measures. Statistical inference results and corresponding key biological insights appear in the Results section, followed by the section Model Validation which briefly discusses the rigor and adequacy of our approach (Technical details that supplement these sections appear in Appendices A–F and the online Supplementary Material). Our paper concludes with an in-depth Discussion section on the biological and practical implications of our integrative modeling approach in the general context of facilitating effective wheat breeding programs via root phenotyping.

## 2. Materials and methods

### 2.1. Data and modeling framework

Each soil core sampled was partitioned in the field into five-centimeter segments from which the number of roots, *y*, was determined every 10 cm up to 180 cm using a fluorescence imaging system (Wasson et al., 2016). Each value of *y* at Depth *t*(= 1, …, *n*_*D*_ where *n*_*D*_ = 18) is the sum of the count imaged from the bottom face of the segment above *t* and that from the top face of the segment below *t* (See Appendix A for details on data collection). Let *y*_*ijkt*_ denote the total number of imaged live roots of Core *k* at Depth *t* for Genotype *i* in Block *j*. Thus, each *i*th genotype is associated with 288 (= *n*_*B*_*n*_*C*_*n*_*D*_) observations of *y* in total. Equivalently, each *t*th depth is associated with 320 (= *n*_*B*_*n*_*C*_*n*_*G*_) observed counts.

**Figure 2:**
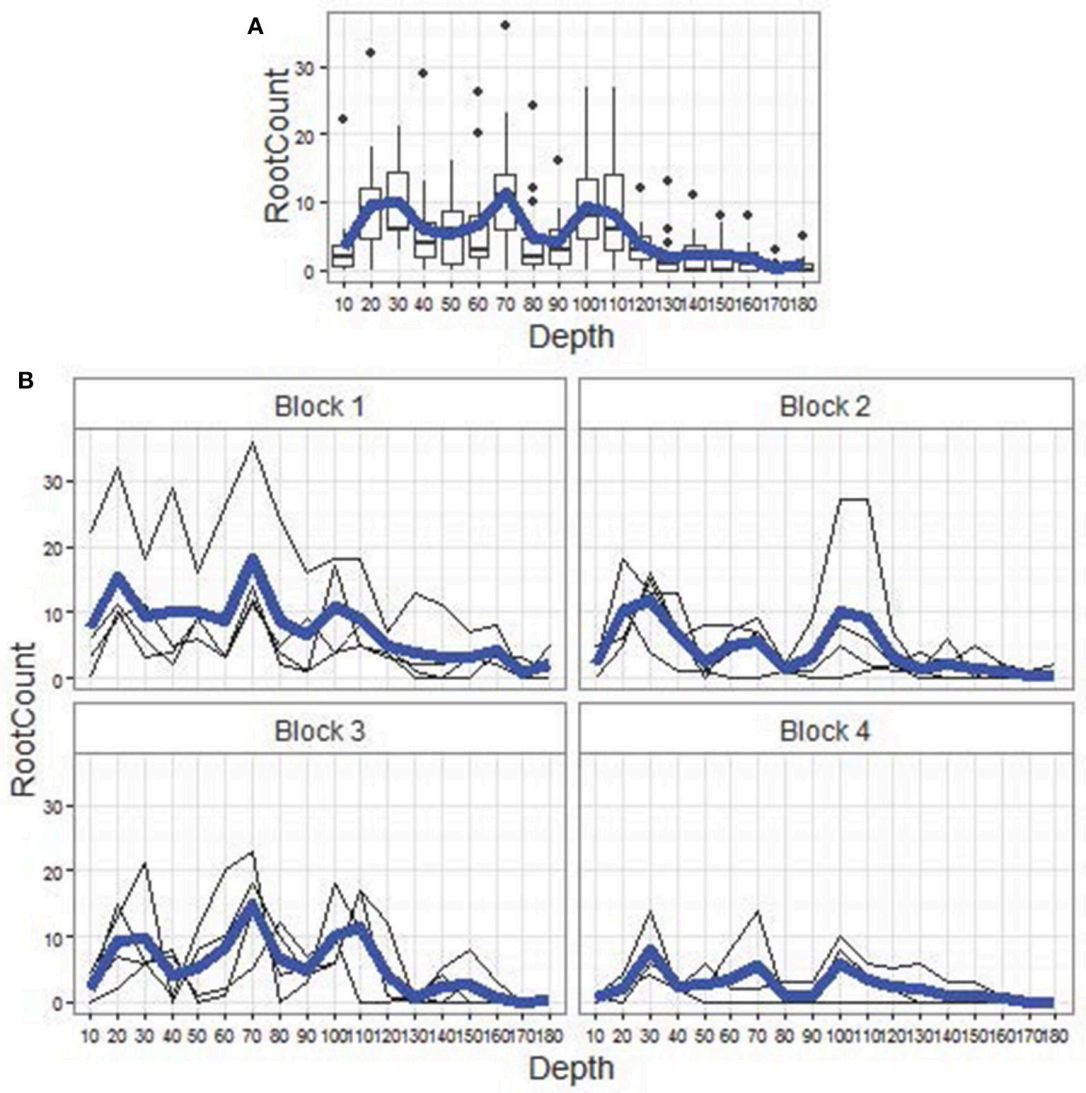
**Data visualizations. (A)** Boxplots of root counts, by depth for genotype G18, pooled across replicate plots (4) and depth-specific core segments (4 per plot). The horizontal axis is depth from 10 cm to 180 cm, at 10 cm intervals. The blue line is the empirical mean root count profile over depth, which, along with the corresponding mean profiles for other genotypes, resembles those in Figure 6 of Wasson et al. (2014). **(B)** Root count profiles (in thin black) over depth, by block (replicate plot, shown as panel label), for genotype G18. Superimposed in bold blue within each block is the within-block empirical mean root count profile.

Data visualizations for Genotype G18 (Figure 2) and other genotypes (not shown) suggest that our observed root counts, *y*, perceivably follow a smooth nonlinear trend over core depth, but subject to substantive noise from the effects of soil physical and chemical properties described above, plus sampling and handling errors. These sources of noise culminate in the profile plots (Figures 2A,B) and associated boxplots (Figure 2A) for *y*. Therefore, a modeling framework comprising the following main model statements was developed to capture the complex noise structure around an idealized smooth trend:

$$\begin{array}{ll} y_{ijkt} & \sim \text{Poisson}\left(\theta_{ij}\left(t\right)\right),\\ \theta_{ij}\left(t\right) & =\psi_{ij}•\gamma_{\alpha_{i}\beta_{i}}\left(t\right)•e^{\phi_{ijt}},\\ \psi_{ij} & =e^{\psi_{0}+\kappa_{j}}e^{\tau_{i}} \end{array}$$

where θ_*ij*_(*t*) denotes the underlying plot-specific Poisson *intensity curve* over depth, i.e., the modeled mean root count at Depth *t* (= 1, 2, …, 18) from the {*i, j*}th plot (for Genotype *i* (= 1, 2, …, 20) observed in Block *j* (= 1, 2, 3, 4)).

**Figure 3:**
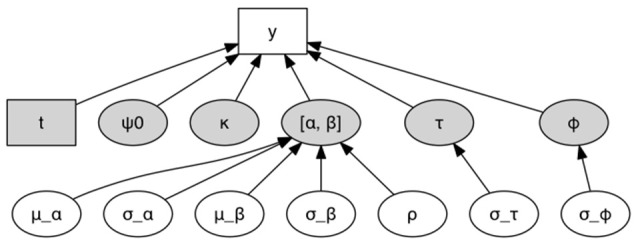
**Hierarchical structure of our modeling framework**. Boxes denote data, and ovals denote model parameters (unobservable). Shaded nodes collectively determine the modeled Poisson intensity, θ.

Intensity θ_*ij*_(*t*) itself is decomposed into fixed and random effects (shaded nodes in Figure 3). Specifically, θ_*ij*_(*t*) comprises a smooth genotype-specific “kernel function,” γ__α__*i*_β_*i*__(*t*), and two sources of multiplicative Gaussian errors: genotype-specific deviation τ_*i*_ and core segment-specific deviation ϕ_*ijt*_. The intensity function's proportionality multiplier ψ_*ij*_, on the logarithmic scale, represents the plot-specific intercept of the {*i, j*}th intensity function. The intercept can be regarded as the modeled mean count (log scale) of the root system just below the soil surface. Therefore, τ_*i*_ corresponds to the genotypic random effect on this near-surface mean count. As such, ψ_*ij*_ itself is random. It is modeled as log-linear, where its mean can be expressed as a study-wide constant ψ_0_ plus a non-random block-specific shift κ_*j*_ (both taken to be fixed effects) (see Appendix B).

### 2.2. The root system's *Bulk* and *Exploration* parameters

**Figure 4:**
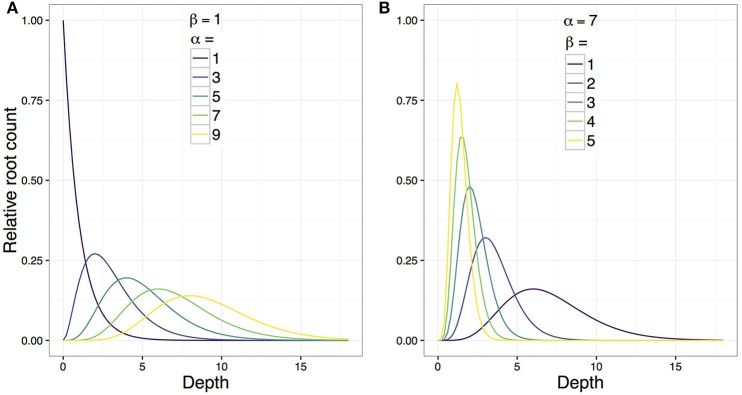
**An illustration of the effect of changing the parameters α_*i*_ and β_*i*_ on the shape of the genotype-specific kernel function (γ__α__*i*_β_*i*__(*t*)) which is proportional to the probability density function of the gamma distribution**. The vertical axis is the idealized relative root count (a dimensionless value). **(A)** Increasing α_*i*_ while fixing β_*i*_(= 1) causes the idealized function's peak to be located deeper under the soil surface and to be less concentrated. Thus, α_*i*_ is a “bulk parameter” that reflects the depth and density of the “bulk” of the root system. **(B)** The effect of increasing β_*i*_ while fixing α_*i*_(= 7) causes an increased skewness in the tail of the idealized function, and consequently decreases the depth of the function's peak from the soil surface and increases its concentration. Hence, β_*i*_ is an “exploration parameter.”

The idealized function $\gamma_{i}\left(t\right)=\gamma_{\alpha_{i}\beta_{i}}\left(t\right)=t^{\alpha_{i}-1}e^{-\beta_{i}t}$ has two genotype-specific parameters, α_*i*_ and β_*i*_, respectively representing the non-negative *shape* and *rate* of the gamma probability density function. Holding β_*i*_ constant and increasing α_*i*_ causes the *i*th kernel function to (a) peak at a lower depth and (b) exhibit more spread around the peak (Figure 4A). Thus, α_*i*_ corresponds to both the depth at which the root system is most dense and its tendency to explore spatially around this depth. Henceforth, we refer to α_*i*_ as the “bulk parameter.”

Similarly, holding α_*i*_ constant and increasing β_*i*_ causes the *i*th kernel function's tail to taper off more quickly, i.e., to exhibit a more slender tail (Figure 4B). Thus, β_*i*_ roughly corresponds to the decline rate of the root system's downward exploration. In other words, the less slender (i.e., fatter) the kernel function's tail, the slower the decline of the root system's downward exploration (or, the bigger the tendency for the root system to explore downwards). Henceforth, we refer to β_*i*_ as the “exploration parameter.”

For each *i*th genotype, parameters α_*i*_ and β_*i*_ are modeled as bivariate log-normal random variables (i.e., they are bivariate Gaussian on the logarithmic scale with unknown correlation ρ). These parameters and the noise terms τ_*i*_ and ϕ_*ijt*_ are each modeled to have a mean that is constant across the study (i.e., not indexed by *i, j, k*, or *t*), and similarly for all the (co)variance parameters in the model (See Appendix B). A visualization of the overall hierarchical structure of our modeling approach appears in Figure 3.

Finally, under the Bayesian inference framework, we specify reasonably non-informative prior distributions to reflect our lack of knowledge, in the absence of data, about the model parameters (see Appendix B). Collectively, the HNLMM and prior distributions as specified above are referred to as *Model 1*. Details on the implementation of *Model 1* appear in Appendix C.

### 2.3. Novel heritability measures

The general notion of heritability is the proportion of phenotypic variation that can be attributed to genetics. Loosely, we have

$$\begin{array}{ll} P\text{henotype} & =G\text{enotype}+E\text{nvironment},\\ \text{heritability} & =\text{Var}\left(G\right)/\text{Var}\left(P\right). \end{array}$$

This definition of heritability assumes that genotypic and environmental variables are independent, linear components of the phenotypic response variable of interest. In practice, the biological notions of phenotype, genotype, and environment are abstract, and their quantifications that can be measured in an experiment may exhibit a complex co-dependence in a nonlinear fashion. Indeed, Moran (1973) pointed out that a quantification of heritability that purely stems from a linear decomposition of the phenotypic response can be nonsensical in practical settings.

In the case that the measurable quantities and experimental design can be reasonably described using Poisson regression, Foulley et al. (1987) adapts the linear (Gaussian) model-based definition of heritability to the scale of the linear predictor in a Poisson regression model, rather than the scale of the phenotypic response. Recently, this definition was extended to a longitudinal Poisson mixed model (Mair et al., 2015). We further extend these ideas to define heritability measures based on segment-level count data.

Our adaptations below emphasize the challenge of detecting trends in root architecture from root count data that are both highly noisy and highly non-Gaussian, and that deviate substantially from a simple Poisson model; while considering data at a reasonably high spatial resolution may mitigate the challenge due to noise, it necessarily requires additional model complexity to address the non-standard statistical behavior of the data, and consequently, a novel quantification of heritability based on our new modeling paradigm.

The formulation of *Model 1* as presented in Appendix B gives rise to a mean number of roots that is nonlinear in its parameters, even on the logarithmic scale. Hence, this mean is not a linear predictor in the usual context of generalized linear models. Nevertheless, at each *t*th depth, we decompose the variability of log θ(*t*) into $\sigma_{\log\theta }^{2}\left(t\right)$ and $\sigma_{\text{genes}}^{2}\left(t\right)$ both of which are spatial in nature.

Here, we must address various aspects of complexity that are non-standard in heritability studies: (1) our analog of Var(*G*), namely, $\sigma_{\text{genes}}^{2}\left(t\right)$, is attributable to the variability of the trio of genotypic parameters τ_*i*_, α_*i*_, and β_*i*_; and (2) it is a spatial function. Thus, it is reasonable to further decompose this Var(*G*) analog into τ-, α-, and β-specific components, as each of the trio pertains to different root architectural features; and the α- and β-specific components are also functions of *t* and are co-dependent except in the naïve case. In Appendix D, we present the four mathematical definitions of heritability (corresponding to $\sigma_{\text{genes}}^{2},\tau ,\alpha$, and β) to handle such complexity.

Finally, we pool depth-specific values by taking the harmonic mean across depths, thus defining a quantity at the genotypic level that summarizes the particular architectural feature across all depths (see Appendix D). The pooling of spatial elements to form an overall heritability measure gives rise to the multiresolution nature of our approach.

## 3. Results

We discuss three major biological insights that arise from the Bayesian inference, i.e., the joint *posterior distribution* among the parameters of *Model 1*.

### 3.1. Root intensity profiles are statistically distinguishable among genotypes

**Figure 5:**
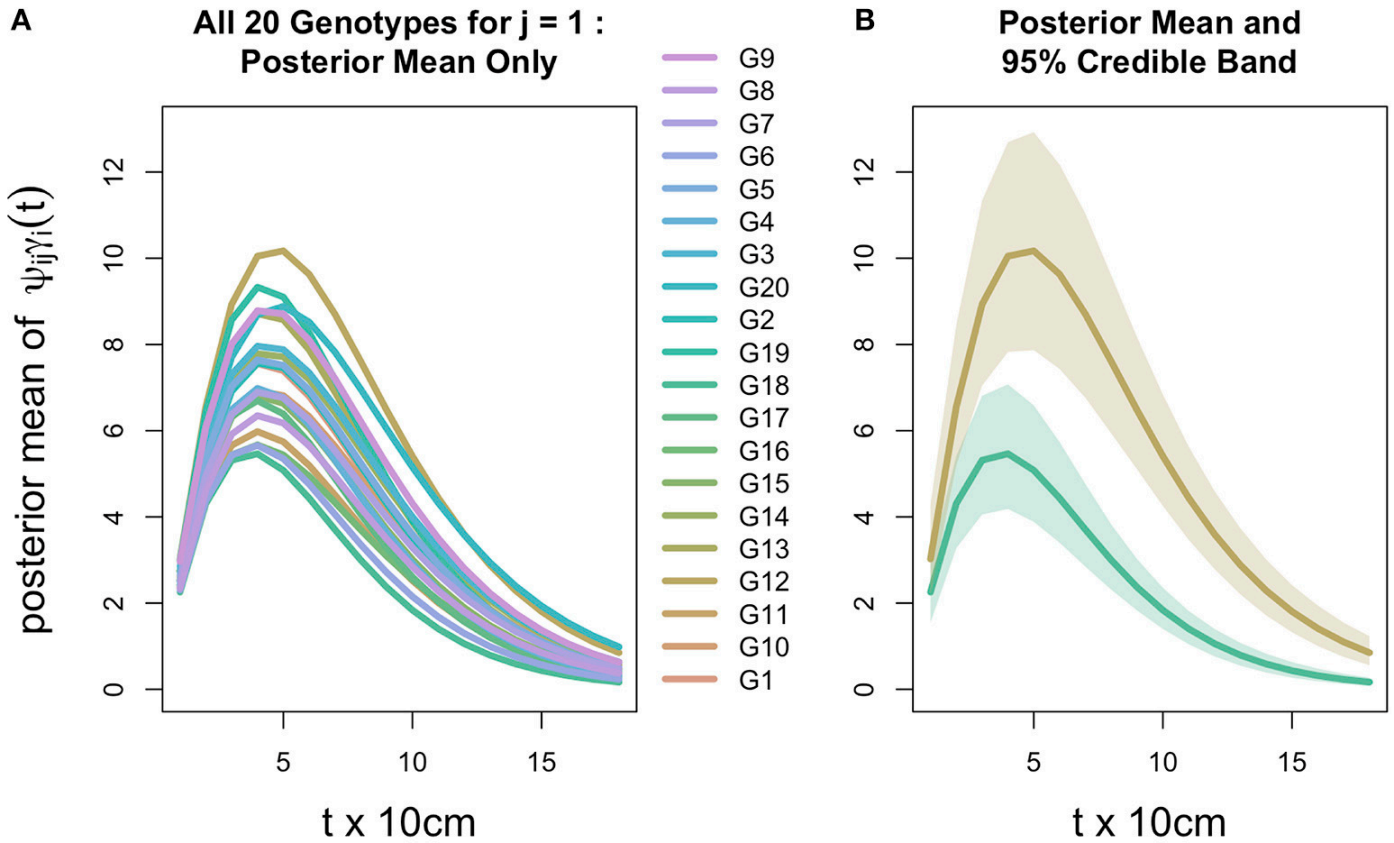
**Inference for root distribution. (A)** Posterior mean (Bayesian estimate) of idealized intensity profile ψ γ(*t*) for replicate block *j* = 1 for all 20 genotypes. Other blocks appear similarly, differing only in the intercept due to the block-specific fixed effect κ in which log ψ is linear. **(B)** Posterior means for Genotypes G12 and G18 (from panel **A**) which are respectively the maximum and minimum curves, each surrounded by a 95% credible band (Bayesian confidence band). Note that credible bands are constructed from depth-wise 95% credible intervals of ψ_*ij*_γ_*i*_(*t*); thus, the lower band limit is constructed by connecting, across the 18 values of *t*, the 2.5th percentiles of the ψ_*ij*_γ_*i*_(*t*) posterior distribution; similarly, the upper band is constructed by connecting the corresponding 97.5th posterior percentiles.

Posterior inference allows us to examine the intensity profiles θ_*ij*_(*t*) and their idealized (denoised) counterparts ψ_*ij*_γ_*i*_(*t*) for any given replicate block *j*. A ψ_*ij*_γ_*i*_(*t*) profile is effectively the intensity profile θ_*ij*_(*t*) but ignoring the random genotype-block interaction ϕ_*ijt*_. Note that ψ_*ij*_ is log-linear in τ_*i*_ and κ_*j*_ without an interaction term. Thus, the behavior among the 20 idealized profiles within any *j*th block is necessarily consistent across all four blocks but for an intercept shift κ_*j*_. Hence, Figure 5 focuses on *j* = 1 to represent the study-wide behavior of the idealized profiles.

All posterior means (Bayesian estimates) of the 20 genotypic idealized profiles ψ_*ij*_γ_*i*_(*t*) are visually distinguishable (Figure 5A); and the study-wide statistical power is very high in determining that the genotypes do not all exhibit the same idealized profile (Figure 5B): 95% credible bands (Bayesian confidence bands) around the maximum and minimum idealized profiles (G12 and G18, respectively) are clearly non-overlapping. (It is analogous to rejecting the null hypothesis in a classical ANOVA at a very low significance level.) This lack of overlap at such a high credible level indicates that, among the 20 genotypes, at least G12 and G18 are highly statistically discernible with respect to their idealized intensity profiles.

**Figure 6:**
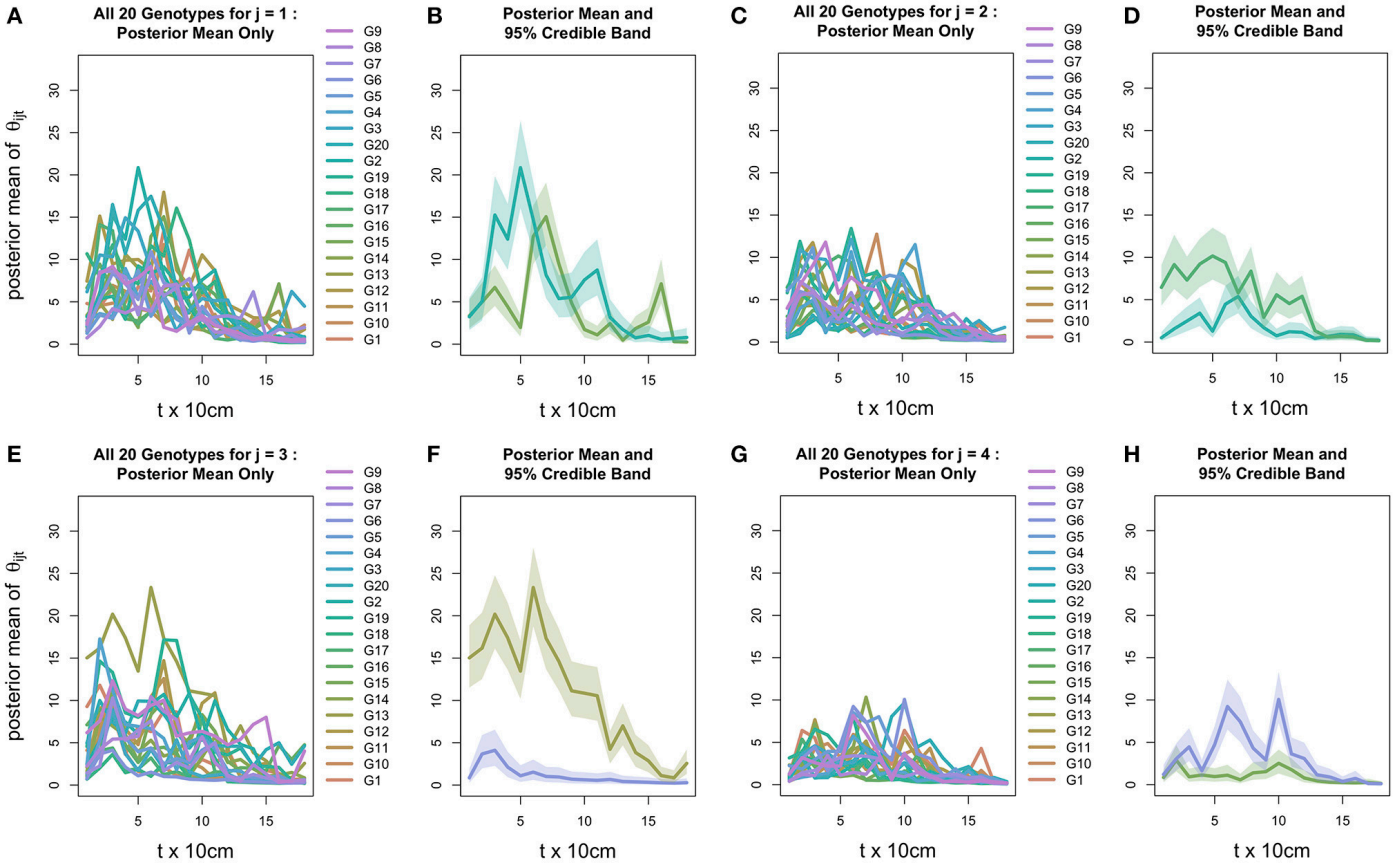
**Posterior mean of intensity profile θ(*t*) for all 20 genotypes, coupled with those genotypes with the maximum and minimum curves and their 95% credible intervals for *j* = 1 (A,B)**, *j* = 2 **(C,D)**, *j* = 3 **(E,F)**, and *j* = 4 **(G,H)**.

While ψ_*ij*_γ_*i*_(*t*) necessarily behaves similarly across all *j*, the genotype-block interaction intrinsic in the plot-specific intensity profile θ_*ij*_(*t*) induces variability in the 20 profiles' collective behavior across *j*, as is evident in Figure 6: in each block, this variability reduces the statistical distinguishability among the 20 genotypes, although in each of Blocks 2, 3, and 4, at least two intensity profiles are highly discernible. Specifically, despite the noisy nature of θ_*ij*_(*t*), Figure 6 shows that in each of Blocks 2–4, at least two intensity profiles θ_*ij*_(*t*) (respectively, (*i* =){*G*2, *G*17} in Block (*j* =)2, {G6, G13} in Block 3, and {G6, G15} in Block 4) are highly statistically discernible due to the general lack of overlap between the pair of block-specific 95% credible bands around θ_*ij*_(*t*).

### 3.2. Root intensity profiles are substantially heritable

**Figure 7:**
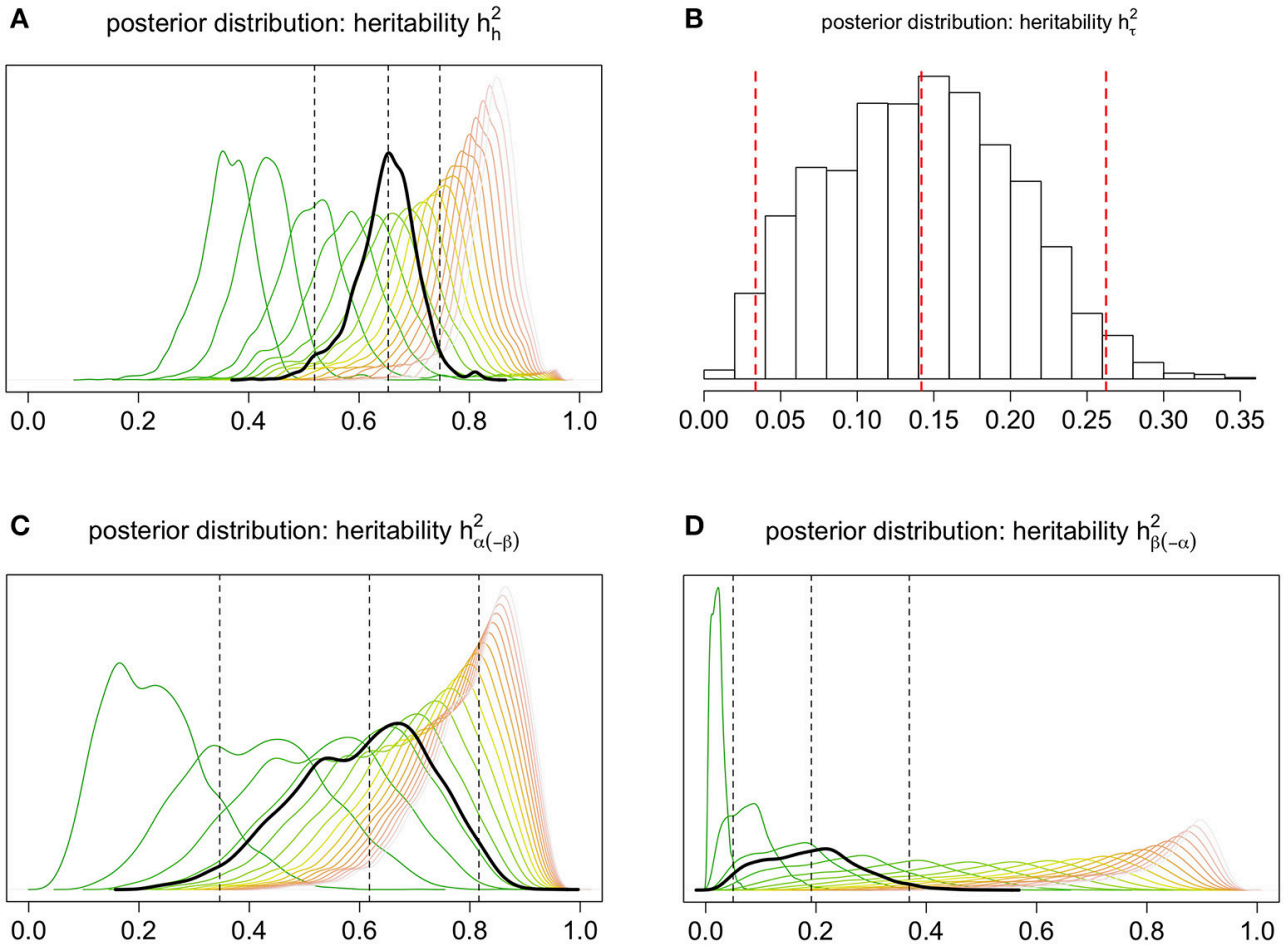
**Posterior distributions of pooled measures of heritability (black), pertaining to (A)** overall root architecture, **(B)** the near-surface intensity, **(C)** the root bulk's location (and size), and **(D)** the root system's decline of penetration; the middle vertical line marks the posterior median (a Bayesian estimate), and the outer lines delimit the 95% credible interval. In **(A,C,D)**, pooling corresponds to integrating depth-dependent heritability over all 18 depths via the harmonic mean of the 18 depth-specific heritability values; the posterior distribution of the unpooled heritability at a given depth is shown in shades of “burnt grass,” where more burnt corresponds to greater depth.

Each of our four genotypic heritability measures is a model parameter that exhibits a posterior distribution, shown in black in Figure 7; three of these are pooled measures, each comprising 18 depth-specific components (Appendix D), shown in color.

**Figure 8:**
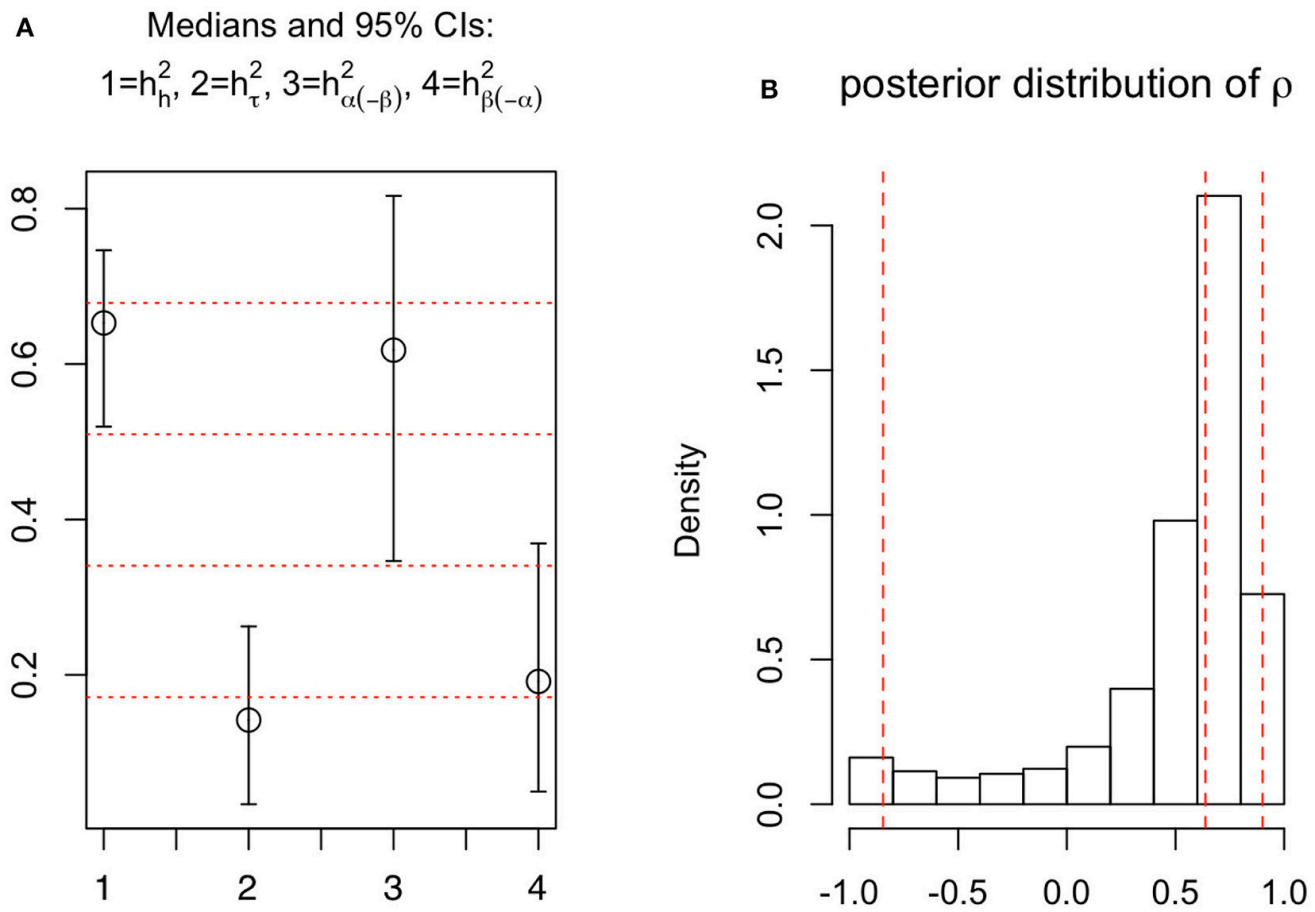
**(A)** 95% credible intervals and posterior medians for the four heritability measures (1 = root architecture; 2 = near-surface mean count, log scale; 3 = bulk parameter; 4 = exploration parameter). **(B)** Posterior distribution of ρ, with vertical lines indicating the 2.5, 50, and 97.5% quantiles.

Focusing on the genotypic level (Figure 7 in black; Figure 8A), the Bayesian estimate and 95% credible interval for heritability of the intensity function are, respectively, 0.65 and (0.52, 0.75); for that of the near-surface mean count they are 0.14 and (0.03, 0.26); the “bulk” parameter, 0.62 and (0.35, 0.82); and the “exploration” parameter, 0.19 and (0.05, 0.37).

Note in Figure 7 that (i) the depth-specific components of each of $h_{h}^{2},h_{\alpha \left(-\beta \right)}^{2}$, and $h_{\beta \left(-\alpha \right)}^{2}$ tend to increase as depth increases, and (ii) the near-surface intensity of root count has low heritability ($h_{\tau }^{2}$). These features of our results indicate that root count features at deeper depths are more heritable than those at shallower depths. In other words, our results provide quantitative rigor for three ideas: the heritability of root architectural traits varies substantively across depth; traits that are associated with a deeper spatial location tend to be more informative about plant genetics; and the depth at which the root system develops its bulk is negatively associated with its tendency to explore deeper. The third notion is further evidenced by another feature of *Model 1*, which is discussed in the next subsection. It is also interesting to note that, although overall heritability $h_{h}^{2}$ is constituted from $h_{h}^{2},h_{\alpha \left(-\beta \right)}^{2}$, and $h_{\beta \left(-\alpha \right)}^{2}$, Figure 8A (which summarizes the genotypic aspects of Figure 7) suggests that each of the latter three tends to be less than $h_{h}^{2}$ itself, thus root architecture on the whole tends to be more heritable than any of these standalone features of the root system.

### 3.3. Linkage exists between near-surface root density development and downward exploration

The modeled correlation, ρ, between the bulk and exploration parameters (both on the log scale), is estimated to be 0.64, with 95% credible interval = (−0.85, 0.90) (see Figure 8B). Due to skewness, the posterior probability for ρ to be positive is 0.88, substantiating that the root system's bulk and downward exploration are generally positively associated architectural features. Specifically, a shallower and more concentrated bulk (small α) is associated with a larger tendency for the root system to explore deeper (small β). This phenomenon may be regarded as “a small β canceling out a large α,” or, the tendency of exploring downwards to exhibit the effect of negating the tendency to develop root density further away from the surface. We elaborate on this discovery under Discussion.

## 4. Model validation

Details of model validation procedures appear in Appendix E. In summary, residual analyses suggest only minor statistical inadequacies of *Model 1*. With respective to the model's predictive performance as measured by the Watanabe-Akaike information criterion (WAIC) (Gelman et al., 2013), its hierarchical structure is essential. Specifically, ignoring the hierarchical structure between the root count intensity function and its various random components that are specific to genotypes, plots, and depths leads to a naïve model that agrees poorly with the empirical behavior of our root count data. Employing the hierarchical structure, the model's predictive performance remains effectively unaffected whether *a priori* dependence between the bulk and exploration parameters is considered; however, we regard this extra dependency as a key biological feature because (a) it improves the interpretability of the model by providing an explicit assessment of the interplay between the bulk and exploration parameters, and (b) this interplay is shown to be substantive based on our field data (as indicated by a smaller *effective number of parameters* for *Model 1* despite its extra complexity due to the *a priori* dependency).

## 5. Discussion

The development of roots in response to the extreme heterogeneity of the soil results in a lack of discernible root system characteristics that can be measured in the field and integrated into crop breeding programs. The inability to breed on the root development of crops and the inadequacy of conventional crop physiological models in addressing the spatial heterogeneity of root systems and soil properties are barriers to the sustainable intensification of agriculture, where root traits are known to be critical to resource-use efficiency and resistance to climatic extremes. Consequently, they are barriers to effective crop yield risk mitigation and food security. Crop physiological models with better predictive ability are much sought after, and novel statistical models can facilitate this pursuit by effectively teasing apart root system physiology from severe heterogeneity. In this paper, we have addressed this knowledge gap by scrutinizing root counts observed using a core-break count method, and by developing a novel modeling approach that accounts for all root count data holistically. Our approach gave rise to new multiresolution heritability metrics, each describing a specific feature of the root count distribution spatially and at the overall genotypic level, which we showed to be substantially heritable. Our integrative approach can allow selective pre-breeding programs for root distribution and may facilitate the identification of genetic markers from field data.

The holistic nature of our approach is an inherent advantage of hierarchical modeling. For model inference, we employed the Bayesian paradigm, which is intrinsically hierarchical in structure. It also has the potential of being greatly flexible: as long as the model is mathematically sound and sufficient computational resources and algorithms are used to implement the model, rigorous statistical inference can be straightforward even for a model with highly complex nonlinear parameters and random quantities that follow non-standard probability distributions. In contrast, classical statistical inference can be too impractical when models or data structures deviate from well-studied scenarios. In our case, the experimental setup and the notion of root architecture together led to a highly non-standard scenario that, under a classical paradigm, would have been much less straightforward to model and subsequently draw inference from. Not only was *y* (the response variable of interest) strictly non-Gaussian, the data were also three-dimensionally spatial in nature, where replicate plots were arranged in a certain 2-dimensional structure (indexed by {*i, j*}), and in turn each plot generating numerous 1-dimensional spatial observations of *y* (indexed by *t*).

Irrespective of the inference paradigm, a caveat of hierarchical modeling is that model complexity in the form of highly nonlinear functional forms and/or intricate hierarchical dependence structures can render the inference so computationally challenging that determining the posterior distribution (for Bayesian inference) or the sampling distributions of estimators and test statistics (for classical inference) would require novel numerical algorithms that are yet to be developed. However, for Bayesian inference in our case, model implementation and model diagnosis/validation were reasonably straightforward to conduct. The satisfactory predictive performance of our *Model 1* (Appendix E) suggests that *Model 1* is scientifically sensible and has yielded biological insights that are superior to what could have been drawn from previous linear models applied to core-level metrics (from collapsing segment-level data).

Although *Model 1* does not account for potential within-core spatial dependency among segment-level root count data (see Appendix F), the biological implications of this model nonetheless will help to define root traits for breeding. The canonical model of root distribution with depth is that of a negative exponential function (Gerwitz and Page, 1974). Gale and Grigal (1987) describe a nonlinear function *Y* = 1 − β^*d*^ where *Y* is the cumulative root fraction from the surface to the depth (*d* cm), and the coefficient β is genotypically determined. This model was later employed by Jackson et al. (1996) to model root distributions across a range of terrestrial biomes.

However, this 1-dimensional model takes no account of the horizontal distance from the base of the plant. It has been observed that root distribution is 1-dimensional with depth in grassland, 2-dimensional in crops planted densely in rows, and 3-dimensional where plants are widely spaced (Bengough et al., 2000). The simulation studies by Grabarnik et al. (1998) showed that root length density—typically the length of root per volume of soil (cm/cm^3^)—for maize decreased nonlinearly with horizontal distance from the stem in the top 40 cm, but below that depth they were homogeneously distributed with horizontal distribution from the plant. Grabarnik et al. (1998) also showed that the roots were subject to clustering at all depths, and that whilst there was no preferential growth in a horizontal plane, the orientation of root growth deviated from the uniform distribution with increasing depth. Similar findings were generated in the simulation study by Bengough et al. (1992), and both studies drew attention to the likely effect of soil structure to further perturb the uniform directional distribution of root development parameters.

The similarity between the model by Gale and Grigal (1987) and the special case of our gamma kernel function where α = 1 should be noted (Figure 4A). Rather than root length density, our model accounts for root counts that are random with respect to sampling position by row in a crop. The model is designed to explain the distribution of root counts with depth at the crop level (and not the plant level). However, the sampling position is likely to have a strong influence on the surface root counts, which explains the low heritability of τ in our model.

Interpreting the biological meaning of the “bulk” and “exploration” parameters (α and β, respectively) is also interesting. In the gamma kernel function, β also affects the depth and intensity of the peak otherwise defined by α (Figure 4). Indeed, our data analysis implicated that α and β were positively correlated (Figure 8B). For our HNLMMs, predictive performance remained largely unaffected whether *a priori* dependence between α and β is considered; however, including this extra dependency improved the interpretability of the model by providing an explicit assessment of the interplay between the root system's tendencies to branch beneath the surface and to explore vertically, deep below the surface.

An explanation for this effect may be found in the structure of the soil; root growth in deeper layers is perceivably constrained to networks of cracks and pores (Gao et al., 2016). White and Kirkegaard (2010) show that in a dense, structured subsoil 85–100% of roots below 60 cm were clumped in pores and cracks in the soil (compared to 30–40% above 60 cm), and 44% of the roots were clumped in pores with more than three other roots. Exploration of the soil for cracks and pores may define the exploitation of the soil by a root system. It has been suggested that plants have evolved randomness and instability in their root system development (Forde, 2009), which may facilitate exploration. The exploration of the shallow layers for cracks and pores may be what determines the eventual depth; our model implies that more branching near the surface gives better access to the subsoil.

The primary purpose of our modeling approach was to distinguish genotypes from root count data that are statistically noisy. The inference for heritability based on the intensity functions suggests that our approach can be used to identify genetic markers of root system distribution in field data; identified markers then could be integrated into breeding programs. The high heritability of the “bulk” parameter also suggests that a breeding program could successfully alter the depth at which a root system proliferates.

Notwithstanding, residual plots (Appendix E and Supplementary Material: Supplementary Figures) suggest some minor statistical inadequacies of *Model 1*. Therefore, it may be advantageous to (1) explicitly model gene-environment interactions (which are implicitly modeled by our current HNLMM due to the marginal dependence among genotypic terms indexed by *i* and environmental terms indexed by *j* and/or *t*); (2) formally model the within-core spatial dependence (possibly at a higher spatial resolution of core depths than the current 10 cm intervals); and (3) also incorporate an additional two-dimensional spatial correlation structure among field plots. In Appendix F, we suggest a possible decomposition at Level 1 of the model hierarchy to address (1), and discuss practical implications of modeling the 3-dimensional spatial dependence to address (2) and (3).

Finally, it may also be of benefit to develop a new quantitative framework to predict root length density from the posterior mean root count profiles while accounting for trials in different soil and climate conditions, under which the response of the intensity functions and their underlying parameters to subsoil constraints could be rigorously exploited.

## Author contributions

AW and GC designed research; AW, GC, AZ, and TB performed research; AW and GC contributed new data processing and analytic tools; GC, AZ, and TB analyzed data; and AW, GC, and AZ wrote the paper.

## Funding

Bayer CropScience (a) funded CSIRO for this research project, part of which was subcontracted to the Australian National University to sponsor GC's involvement after June 2015; and (b) sponsored the CSIRO Agriculture Vacation Scholarship 2014 that was held by TB, under which he contributed to the basic model that was later extended to the approach in this paper.

### Conflict of interest statement

The authors declare that the research was conducted in the absence of any commercial or financial relationships that could be construed as a potential conflict of interest.

## Appendix A

### Data collection

The field trial was conducted at Ginninderra Experiment Station in Canberra, Australia (35°12′29.0″S 149°04′59.0″E), from late May to late December 2013 (the typical wheat growing season for the region) in alluvial cracking clay plots that were 1.3 m long. Twenty spring wheat genotypes (anonymized in this paper) were drawn from a collection of standard cultivars and from a multigenic mapping population on the basis of prior experimentation on root distributions in the field; each was sown with a tractor-drawn plot seeder in ten rows spaced 18 cm apart in a randomized block design with four replicated blocks of plots (Figure 1A). A seed was sown roughly 3 cm apart in each row; the final sowing density was ~150 plants/m^2^. A fertilizer (N:P:K:S = 14:12.7:0:11) was applied at 120 kg/ha at sowing, with urea added for additional N during the growing season. Prophylactic fungicide and herbicide treatments were applied to the trial to suppress weeds and prevent disease. In early January 2014 after the trial had matured and been harvested, four soil cores of ~1.8 m in length were collected from each plot using 2 m long, 42 mm diameter stainless steel coring tubes driven into the soil vertically with a tractor-mounted hydraulic push press (Wasson et al., 2014). Our field sampling technique ensured that within each plot the cores were reasonably independent of each other (Figure 1B). Each core was *broken* into segments rather than *sliced*, so that the roots traversing the plane of the break would emerge intact from one of the two broken faces; the same root could not be visibly intact on both faces simultaneously. (Slicing the roots would have left only the cross sectional area on the face: 50–150 microns in diameter and difficult to detect.) Hence, the root counts on the adjoining faces can be regarded as independent values which, when combined to form *y*, represent the number of roots traversing the break plane at that depth. The fluorescence imaging system generates root counts (Wasson et al., 2016) which necessarily differ from an observer's manual counts, although both are subject to measurement error. The raw imaging data were processed (available from Supplementary Material: Dataset) and visualizations produced with the statistical programming language R (R Core Team, 2015) using the packages “dplyr” (Wickham and Francois, 2015) and “ggplot2” (Wickham, 2009).

## Appendix B

### Formulation of *Model 1* and variants

Recall that *y*_*ijkt*_ denotes an observed root count, where {*i, j, k, t*} indexes {genotype, block, core, depth}, for *i* ∈ {1, 2, …, 20}, *j* ∈ {1, 2, 3, 4}, *k* ∈ {1, 2, 3, 4}, and *t* ∈ {1, 2, …, 18}. Taking $\gamma_{i}\left(t\right)=t^{\alpha_{i}-1}e^{-\beta_{i}t}$ which is the kernel of the gamma probability density function and letting “N” and “BVN,” respectively denote “normal” and “bivariate normal,” our model statements can be rewritten as follows:

$$\text{Level }1:\{\begin{array}{c} \left[y_{ijkt}|\theta_{ijt}\right]\sim \text{Poisson}(\text{mean}=\theta_{ijt}),\\ \log\theta_{ijt}=\psi_{0}+\kappa_{j}+(\alpha_{i}-1)\log t-\beta_{i}t+(\tau_{i}+\phi_{ijt}); \end{array}$$

$$\text{Level }2:\{\begin{array}{c} \;\left[\log\alpha_{i},\log\beta_{i}\right]\prime |\mu ,\Sigma \sim \text{BVN}(\mu ,\Sigma ),\\ \left[\phi_{ijt}|\sigma_{\phi }^{2}\right]\sim N(\text{mean}=0,\text{var}=\sigma_{\phi }^{2}),\;\left[\tau_{i}|\sigma_{\tau }^{2}\right]\\ \;\sim N(0,\sigma_{\tau }^{2}) \end{array}$$

where

$$\mu =\left[\begin{array}{c} \mu_{\alpha }\\ \mu_{\beta } \end{array}\right],\;\Sigma =\left[\begin{array}{cc} \sigma_{\alpha }^{2} & \rho \sigma_{\alpha }\sigma_{\beta }\\ \rho \sigma_{\alpha }\sigma_{\beta } & \sigma_{\beta }^{2} \end{array}\right];$$

and κ_*j*_s are fixed effects that require a linear constraint to ensure model identifiability: we take κ_4_ = 0. (See Figure 3 for a schematic of the two levels of our HNLMM.)

For Bayesian inference, prior distributions are required for all fixed-effects parameters κ_*j*_s, ψ_0_, μ_α_, μ_β_, and (co)variance parameters $\rho ,\sigma_{\alpha }^{2},\sigma_{\beta }^{2},\sigma_{\tau }^{2}$, and $\sigma_{\phi }^{2}$. To reflect our lack of *a priori* insight (in the absence of data) into the likely values of these parameters, each was given a standard diffuse prior: the Fisher-transformation *arctanh*(ρ) and fixed effects were all assumed to be independent zero-mean Gaussians with a variance of 10^4^, and the variance parameters were assumed to follow independent and identical inverse-gamma distributions with values 1 and 0.1, respectively, for the shape and rate parameters. The resulting Markov chains of posterior draws exhibited very poor mixing for *Model 1* (as well as *Model 2*, obtained by prespecifying ρ = 0) when a smaller rate parameter value, namely, 0.01 or 10^−4^, was used. As smaller rate parameter values correspond to more diffuse inverse-gamma priors, the poor mixing suggests mildly weak identifiability (even for the smaller *Model 2*). This also suggests that to improve inferential power for and the identifiability of *Model 1*, one could conduct a future field study that consists of a larger number of plots and/or depths, and/or employ stronger priors based on the inference we have presented in this current article.

For model validation (Appendix E), we also considered smaller models: *Model 2* by prespecifying ρ = 0 in *Model 1*; and *Model 3* by taking all *Model 2* parameters (including those that are genotype-specific) to be fixed effects.

**Remarks**. Note that alternative parametrizations of depth are possible. Under Supplementary Material: Model Parametrization, we discuss the so-called *canonical scale* for depth, on which the statistical inference is invariant to certain reparametrizations including the conventional *t* scale (i.e. 1, …, *n*_*D*_) in this paper. This invariance is similar to that of Chiu and Lockhart (2010), where the rigor of the statistical inference is developed on the canonical scale rather than the conventional scale.

## Appendix C

### Implementation of *Model 1* and variants

Bayesian inference requires the derivation of the joint posterior distribution of all model parameters. In our case, this distribution is intractable and Markov chain Monte Carlo (MCMC) was used to approximate it. For this, we used the RStan MCMC software (R Core Team, 2015; Stan Development Team, 2016) to fit *Model 1* and its variants for model refinement purposes (see Supplementary Material: Computer Code). Of the 320 cores sampled, nine were ignored as they failed to yield root count data. While missing data can be imputed under extra model assumptions, the Stan framework did not yet readily allow simulation of discrete parameters, and thus imputation of the missing root counts was not performed.

## Appendix D

### Multiresolution heritability under *Model 1*

To handle the non-linearity—even on the logarithmic scale—of the mean number of roots θ in *Model 1*, we decompose the variability of log θ(*t*) into

$$\sigma_{\log\theta (t)}^{2}=\sigma_{\text{genes}}^{2}(t)+\sigma_{\phi }^{2}$$

where

$$\begin{array}{l} \sigma_{\text{genes}}^{2}(t)=\sigma_{\tau }^{2}+{\left(\log t\right)}^{2}(e^{\sigma_{\alpha }^{2}}-1)e^{2\mu_{\alpha }+\sigma_{\alpha }^{2}}+t^{2}(e^{\sigma_{\beta }^{2}}-1)e^{2\mu_{\beta }+\sigma_{\beta }^{2}}\\ \;-(t\log t)(e^{\rho \sigma_{\alpha }\sigma_{\beta }}-1)e^{\mu_{\alpha }+\mu_{\beta }+(\sigma_{\alpha }^{2}+\sigma_{\beta }^{2})/2} \end{array}$$

is attributable to the variability of the trio of genotypic parameters τ_*i*_, α_*i*_, and β_*i*_, while the study-wide parameter $\sigma_{\phi }^{2}$ is attributable to the pure noise term ϕ_*ijt*_. Note that the parameters $\sigma_{\tau }^{2},\sigma_{\alpha }^{2},\sigma_{\beta }^{2},\mu_{\alpha },\mu_{\beta }$, and ρ stipulate the collective statistical behavior, *a priori*, of τ, α, and β.

As such, we define four different measures of heritability, namely, $h_{h}^{2},h_{\alpha \left(-\beta \right)}^{2},h_{\beta \left(-\alpha \right)}^{2}$, and $h_{\tau }^{2}$, each at the genotypic level, by letting

$$\begin{array}{l} h_{h}^{2}(t)=\frac{\sigma_{\text{genes}}^{2}(t)}{\sigma_{\text{genes}}^{2}(t)+\sigma_{\phi }^{2}}\\ \;=\text{ depth-specific heritability of intensity function at }t,\\ \;h_{h}^{2}=\text{heritability of overall architecture}\\ \;=\text{harmonic mean of }h_{h}^{2}(t)=T/\sum_{t=1}^{T}\left(1+\frac{\sigma_{\phi }^{2}}{\sigma_{\text{genes}}^{2}(t)}\right);\\ h_{\alpha (-\beta )}^{2}=\text{heritability of root bulk}\prime \text{s location }(\text{and size})\text{ on log}\\ \text{ scale, ignoring its relation with penetration rate}\\ \;=\text{harmonic mean of }\\ \;\left\{\frac{{\left(\log t\right)}^{2}(e^{\sigma_{\alpha }^{2}}-1)e^{2\mu_{\alpha }+\sigma_{\alpha }^{2}}}{{\left(\log t\right)}^{2}(e^{\sigma_{\alpha }^{2}}-1)e^{2\mu_{\alpha }+\sigma_{\alpha }^{2}}+\sigma_{\phi }^{2}}\text{ for }t>1\right\};\\ h_{\beta (-\alpha )}^{2}=\text{heritability of root's decline rate of penetration on log}\\ \text{ scale, ignoring its relation with bulk location}\\ \;=\text{harmonic mean of }\left\{\frac{t^{2}(e^{\sigma_{\beta }^{2}}-1)e^{2\mu_{\beta }+\sigma_{\beta }^{2}}}{t^{2}(e^{\sigma_{\beta }^{2}}-1)e^{2\mu_{\beta }+\sigma_{\beta }^{2}}+\sigma_{\phi }^{2}}\right\};\\ \;h_{\tau }^{2}=\frac{\sigma_{\tau }^{2}}{\sigma_{\tau }^{2}+\sigma_{\phi }^{2}}\;=\text{heritability of intensity function's intercept}\\ \text{ on log scale}. \end{array}$$

Note that each of $h_{h}^{2},h_{\alpha \left(-\beta \right)}^{2}$, and $h_{\beta \left(-\alpha \right)}^{2}$ comprises depth-specific heritability components, but $h_{\tau }^{2}$ does not (and thus, its definition does not require the use of the harmonic mean).

## Appendix E

### Validating *Model 1*

#### (a) Predictive performance

Although more complex models typically follow the data more closely, they may have poorer predictive performance due to potential overfitting. We consider the predictive performance of *Model 1* by comparing its value of the WAIC to those for the simpler *Models 2* and *3*, both nested within *Model 1*. The WAIC is a measure of a model's predictive accuracy, and it is asymptotically equivalent to the leave-one-out cross-validation method, the latter of which addresses the notion of the mean squared error but requires substantive computational effort for a dataset as large as ours (Vehtari and Gelman, 2014). In contrast, the WAIC can be easily computed as part of the MCMC implementation of the Bayesian inference (see Supplementary Material: Computer Code).

As mentioned in Appendix B, the modeled correlation between the bulk and the exploration parameters on the log scale was prespecified as ρ = 0 in *Model 2*; *Model 3* considers all model parameters (including those that are genotype-specific) as fixed effects by naïvely prespecifying

$$\begin{array}{l} \sigma_{\phi }^{2}=\tau_{20}=\kappa_{4}=\mu_{\alpha }=\mu_{\beta }=\rho =0,\\ \text{ prior Var}(\psi_{0})=\sigma_{\tau }^{2}=\sigma_{\kappa }^{2}=25,\\ \;\sigma_{\alpha }^{2}=\sigma_{\beta }^{2}\;3. \end{array}$$

Note, that the values 3 and 25 for the prior variance of ψ_0_, τ_*i*_, κ_*j*_, α_*i*_, or β_*j*_ constitute informative prior distributions for these fixed effects. Before considering 3 or 25, we had specified 10^3^ or 10^4^ for diffuseness. However, in either case, *Model 3* failed to converge due to a weakly identifiable α_8_. Consequently, we decided to employ the more restrictive (but defensibly so) prior variances of 3 and 25 according to the following argument.

Based on Weaver (1926), we deduce that for a wheat plant, the total number of roots at any given depth has a magnitude that is *o*(100), and thus a generous approximation for the standard deviation (SD) of θ_*ijt*_ is 100, or for SD(log θ_*ijt*_) is log 100. For *Model 3*, note that

$$\text{E}(\log\theta_{ijt})=\psi_{0}+\tau_{i}+\kappa_{j}+(\alpha_{i}-1)\log t-\beta_{i}t$$

where the sets {τ_*i*_}, {α_*i*_}, {β_*i*_}, and {κ_*j*_} each follows a linear constraint. Thus, for the priors of the fixed effects, heuristically we let

$$\begin{array}{l} \left(\sqrt{25}>)\;\log\;100=\text{SD}(\psi_{0})=\text{SD}(\tau_{i})=\text{SD}(\kappa_{j}\right)\\ \;(\sqrt{3}>)\;\log\;\log\;100=\text{SD}(\log\;\alpha_{i})=\text{SD}(\log\;\beta_{i}) \end{array}$$

for all *i* ≠ 20 and *j* ≠ 4.

For each of *Models 1–3*, we computed the WAIC based on Vehtari and Gelman (2014) (see Supplementary Material: Computer Code); they appear in Table A1. There, one can see that predictive performance improved drastically (WAIC decreased from > 46000 to < 35000) from the naïve fixed-effects *Model 3* to the mixed-effects *Models 1* and *2*, both of which are much more complex. Between the two complex models, although *Model 1* is slightly larger than *Model 2* (by a single parameter that represents *a priori* dependence between the bulk and exploration parameters), effectively they perform equally well in predictive power, as suggested by a merely nominal difference (=3) in WAIC values.

**Table A1:** **Values of the Watanabe-Akaike information criterion (WAIC) as a measure of predictive performance by our Bayesian HNLMMs**.

**Model**	**Description**	**WAIC**
*1*	Most sophisticated among our models, as presented in this paper	34198
*2*	Same as *Model 1*, but with a prespecified ρ = 0	34195
*3*	Naïve preliminary model: same as *Model 2*, except with ϕ_*ijt*_ term missing and all of ψ_0_, τ_*i*_, α_*i*_, β_*i*_, and κ_*j*_ taken as fixed effects	46627

*A smaller WAIC value suggests better model performance*.

In addition to a mere nominal difference in WAIC values between *Models 1* and *2*, we also observed that the approximate values for the effective number of parameters, p_WAIC_ (used in the computation of WAIC), increased from 4011 for *Model 1* to 4014 for *Model 2*. Importantly, although model complexity was increased from *Model 2* to *Model 1* by correlating the genotypic bulk and exploration parameters through a study-wide parameter, the additional parameter reduced the models overall amount of unknownness. Thus, these values of *p*_WAIC_, along with those of the WAIC and the substantively large posterior probability that ρ > 0, suggest the merit of retaining ρ as an unknown parameter in the HNLMM.

#### (b) Residual plots

Next, we inspect violin plots produced by the R package “vioplot” (Adler, 2005) for residuals that correspond to the Level 1 noise terms τ_*i*_ and ϕ_*ijt*_ in *Model 1*. Non-noise-like patterns in these residual plots would suggest the statistical inadequacy of *Model 1* for our data.

Figure S1 is based on the posterior distribution of ϕ_*ijt*_ (posterior median shown as black dot), plotted against the posterior mean of log θ_*ijt*_ rather than the log-transformed ${\overset{-}{y}}_{ij+t}=\underset{k}{\sum }y_{ijkt}/4$. This is because $\overset{-}{y}$_*ij*+*t*_ = 0 for 181 out of all 1440 combinations of {*i, j, t*} (see Section Discussion for possible implications). Figure S1 shows that ϕ_*ijt*_ has a (a) slight tendency to increase with log θ_*ijt*_, and (b) a distinctive non-random relationship with log θ_*ijt*_ especially when the latter is small (which is typically at lower depths). We break down this relationship by the residual violin plots in Figures S2–S6.

Overall, we observe the following minor anomalies:

- τ_*i*_ vs. $\overset{-}{y}$_*i*++*t*_ (Figure S3): at many depths *t*, the random effect τ_*i*_ has a slight tendency to be negative for small observed values of $\overset{-}{y}$_*i*++*t*_, and positive for large observed values of $\overset{-}{y}$_*i*++*t*_;
- ϕ_*ijt*_ vs. *i* (Figure S4): at *t* = 160, 170, or 180 cm, the residual ϕ_*ijt*_ for a small number of plots ({*i, j*} combinations) has a tendency to be highly positive;
- ϕ_*ijt*_ vs. *t* (Figure S5): for the 3rd replicate block (*j* = 3), many genotypes (e.g., *i* = 2, 12, 14, etc.) at the deepest six depths are associated with ϕ_*ijt*_ that increases with depth systematically; the same applies to *j* = 4 and *i* = 8, 9, 11, etc.; additionally, the plot {*i, j*} = {5, 3} shows that ϕ_*ijt*_ has a tendency to be all positive;
- ϕ_*ijt*_ vs. *j* (Figure S6): the same conclusion as for Figure S4 for *t* = 160, 170, or 180 cm; additionally, ϕ_*ijt*_ for a small number of plots has a slight tendency to be positive at *t* = 30 or 100 cm.

Altogether, the residual violin plots suggest that the statistical inadequacy of *Model 1* lies in the modeled behavior of θ_*ijt*_ across the deeper depths for specific combinations of {*i, j*}. Under Section Discussion, we provide an overview of possible directions that may be taken to improve the adequacy of our HNLMM.

## Appendix F

### Some limitations and possible extensions

Note, that while 5 cm segments were produced in the field, the first, third, fifth, etc. depths were ignored in the statistical modeling; only imaged counts at depths in 10 cm increments from the surface were considered. This implies that within the same core, the resulting counts *y* were less spatially autocorrelated due to a lower spatial resolution of the data from omitting alternate segments from consideration. With segment-level counts *y* thus produced, we considered for statistical modeling 18 (= *n*_*D*_) depth values from each core, from depth 10 cm to depth 180 cm.

We now discuss possible extensions to *Model 1*. Although the hierarchical structure of our HNLMM already addresses some over- or under-dispersion in the raw root counts, an abundance (181/1440 > 12%) of observed zero-mean root counts (averaged over four replicate cores) suggests a potential need to include a formal zero-inflation component in a future improved model (e.g., via a mixture model). Furthermore, at present our HNLMM does not include formal (a) gene-environment interactions or (b) spatial statistical modeling (Gelfand et al., 2010) of within-core spatial dependency among segment-level root count data at a 10 cm spatial resolution. For (a), it is possible to further decompose $\phi_{ijt}=\phi_{ij}^{*}+\phi_{it}^{**}+\phi_{ijt}^{***}$, whereby $\phi_{ij}^{*}$ and $\phi_{it}^{**}$ are explicit gene-environment interaction terms at Level 1 of the model hierarchy, and $\phi_{ijt}^{***}$ is the pure noise term. For (b), spatial statistical modeling would impose substantial complexity to the statistical inference and computational burden. However, the residual plots perhaps suggest that formal spatial modeling could be a valuable additional component for our HNLMM, especially with data at the 5 cm resolution. A possible spatial structure could be an autoregressive dependence over depth, and/or a nearest-neighbor dependence (among field plots) that constitutes a Markov random field (Gelfand et al., 2010).
