# Supplementary material for: Differentiating Wheat Genotypes by Bayesian Hierarchical Nonlinear Mixed Modeling of Wheat Root Density
Source: Front Plant Sci. 2017 Mar 2;8:282. doi: 10.3389/fpls.2017.00282 (PMC5332416; doi:10.3389/fpls.2017.00282)
Supplement: Supplementary file 1 [file DataSheet1.pdf]

Supplementary Material: Differentiating wheat genotypes by Bayesian hierarchical nonlinear mixed modeling of wheat root density

Anton P. Wasson*, Grace S. Chiu*, Alexander B. Zwart, and Timothy R. Binns
*Correspondence:
Anton P. Wasson:
anton.wasson@csiro.au

Grace S. Chiu:
grace.chiu@anu.edu.au

1 DATASET

See file ‘`Data Sheet 2.XLSX`’ (Excel workbook) at `http://journal.frontiersin.org/article/10.3389/fpls.2017.00282/full#supplementary-material`.

2 COMPUTER CODE

See file ‘`Data Sheet 3.zip`’ (zipped tar archive) at `http://journal.frontiersin.org/article/10.3389/fpls.2017.00282/full#supplementary-material`.

3 MODEL PARAMETRIZATION

Note that our mathematical expression of *Model 1* and its variants employs a convention for longitudinal analyses in which depth t (an analog of time) is effectively an integer index ranging over 1 to 18. In practice, an alternative parametrization may be preferred, such as $t = 10, 20, ..., 180$ cm, or even to mathematically map the observed spatial domain to the unitless interval (0, 1] so that the labels are $t = 1/T, 2/T, ..., (T-1)/T, 1$ where T is the maximum number of core segments. We refer to the latter as the *canonical scale* for depth, for which we discuss as follows the invariance of our model inference whether the depth scale employed in practice is canonical or otherwise.

In general, as long as the root counts are observed at regular spatial intervals along a soil core, the set of labels

$$s_{t,c} = ct \in \{c, 2c, ..., Tc\}$$

is possible in practice for some $c \neq 0$. However, do different values of c result in different statistical inference?

The answer is "no." Note that the intensity function's kernel is

$$\begin{aligned}\gamma(t) &= t^{\alpha-1}e^{-\beta t}\\ &= \frac{1}{c^{\alpha-1}}(ct)^{\alpha-1}e^{-\beta tc/c}\\ &= \frac{1}{c^{\alpha-1}}s_{t,c}^{\alpha-1}e^{-\beta s_{t,c}/c} \qquad \equiv \gamma^*(s_{t,c}).\end{aligned}$$

We define the canonical scale for depth as $s_{t,c=1/T}$, so that

$$s_t = s_{t,c=1/T} \in \{1/T, 2/T, ..., (T-1)/T, 1\} \in (0, 1].$$

Thus, on the canonical scale, the formulation of *Model 1* remains the same except for

$$\begin{aligned}\log\theta_{ijt} &= \log\psi_{ij} + \log\gamma^*(s_t;\alpha_i,\beta_i) + \phi_{ijt}\\ &= \psi_0 + \tau_i + \kappa_j + (\alpha_i - 1)\log(Ts_t) - T\beta_i s_t + \phi_{ijt}.\end{aligned}$$

This reparametrization using γ^* is linear in both s_t and $T = 1/c$, and thus the statistical inference is invariant to any of the conventional depth scale t, the reparametrized scale involving a non-zero c, or the canonical scale s_t.

4 SUPPLEMENTARY FIGURES

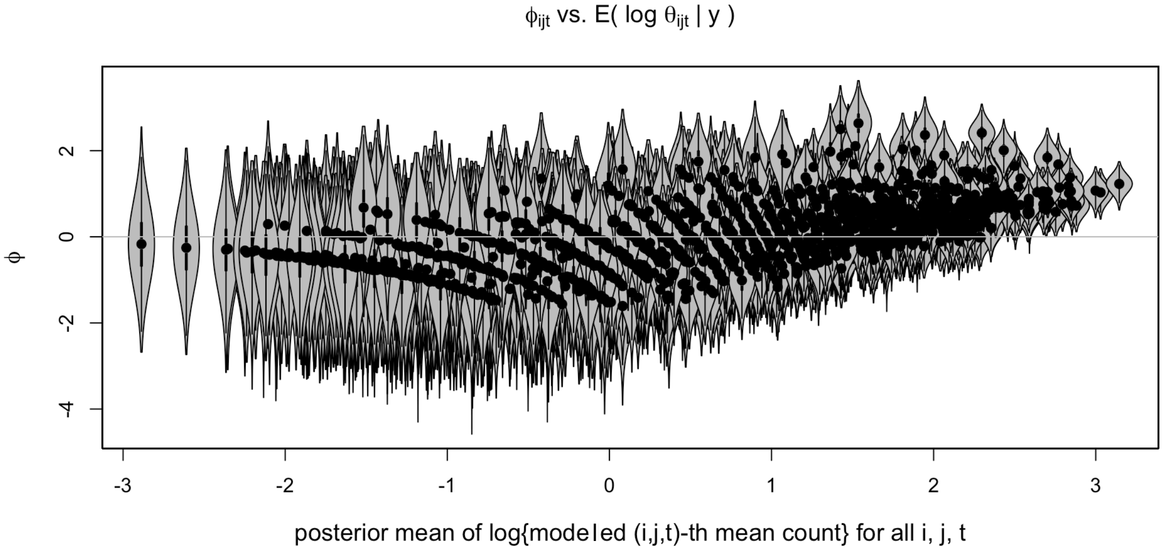

Figure S1. Violin plots: Level 1 noise ϕ against the posterior mean of log(modeled mean root count), for all $\{i, j, t\}$ combinations. The median that corresponds to each $\{i, j, t\}$-th violin of ϕ is shown in black. Some non-random patterns are noticeable.

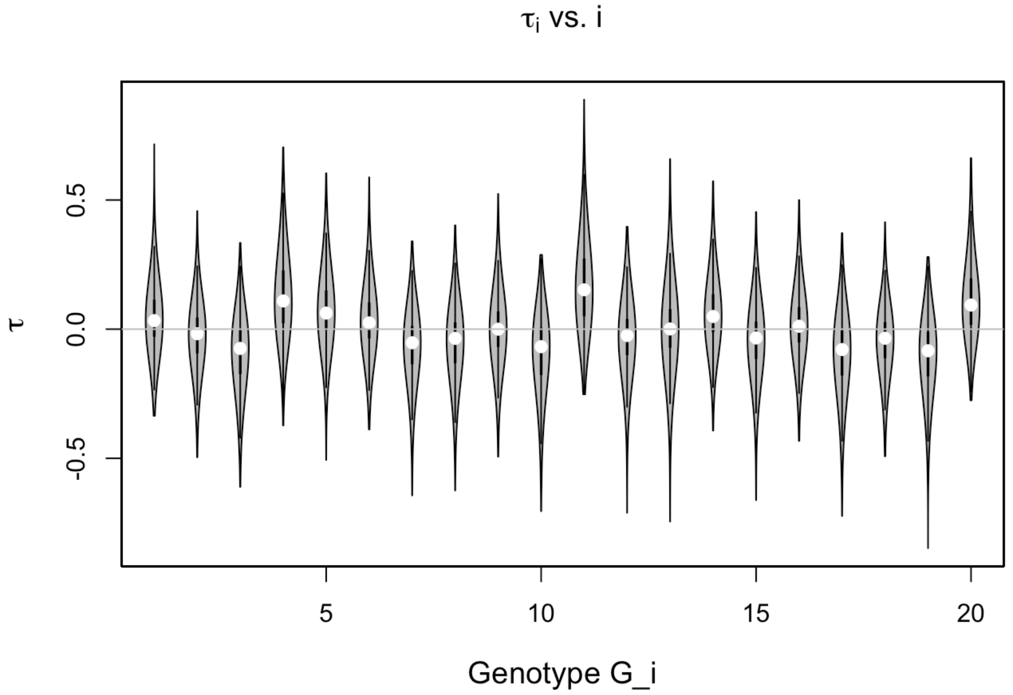

Figure S2. Violin plots: genotypic random effect τ against genotype i. Each ith violin also shows the posterior median of τ in white. No anomalies are apparent.

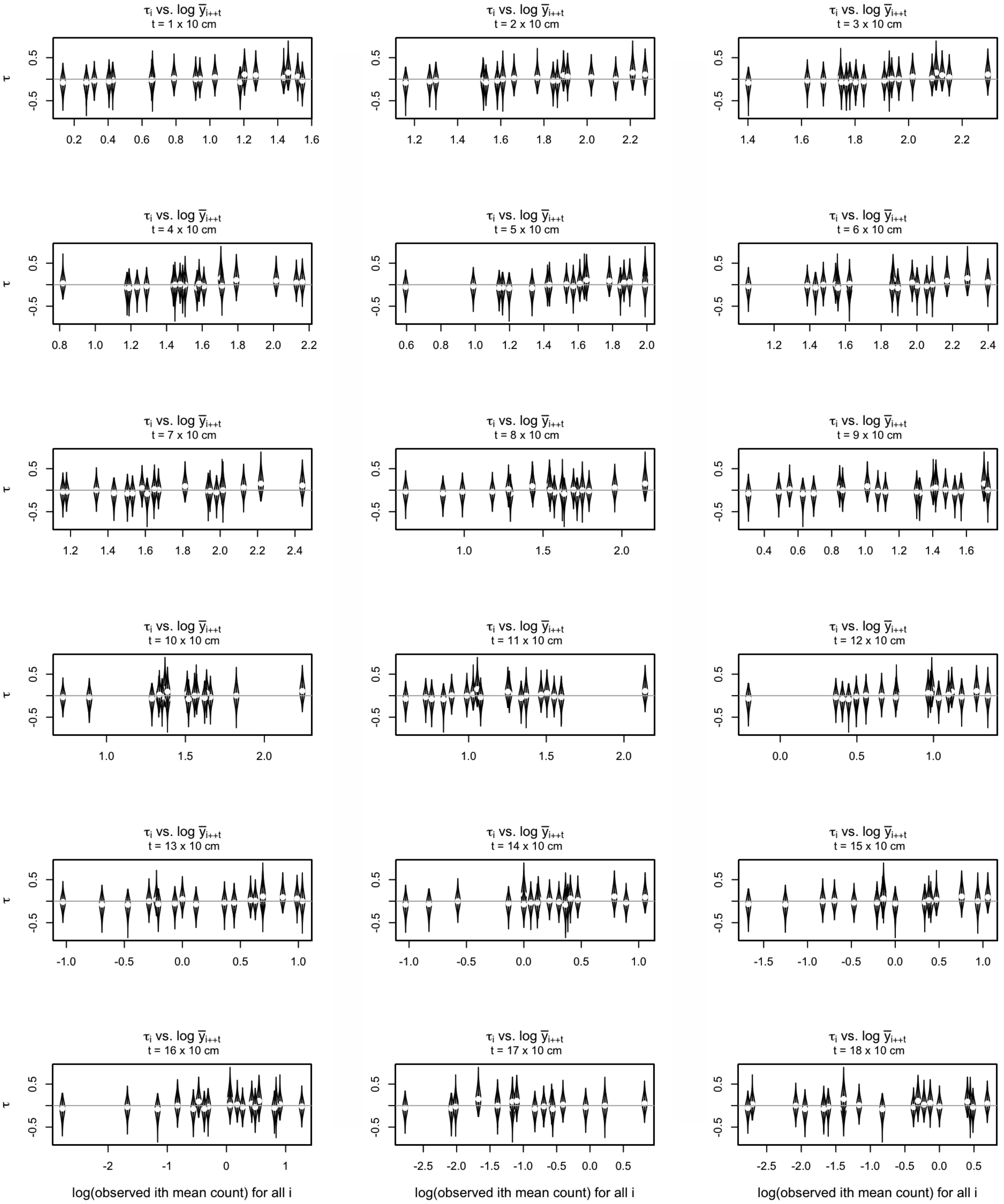

Figure S3. Violin plots: random effect τ against log(mean count); each panel is associated with a particular depth t. Posterior medians of τ are shown in white. A slight increasing trend is noticeable at various values of t.

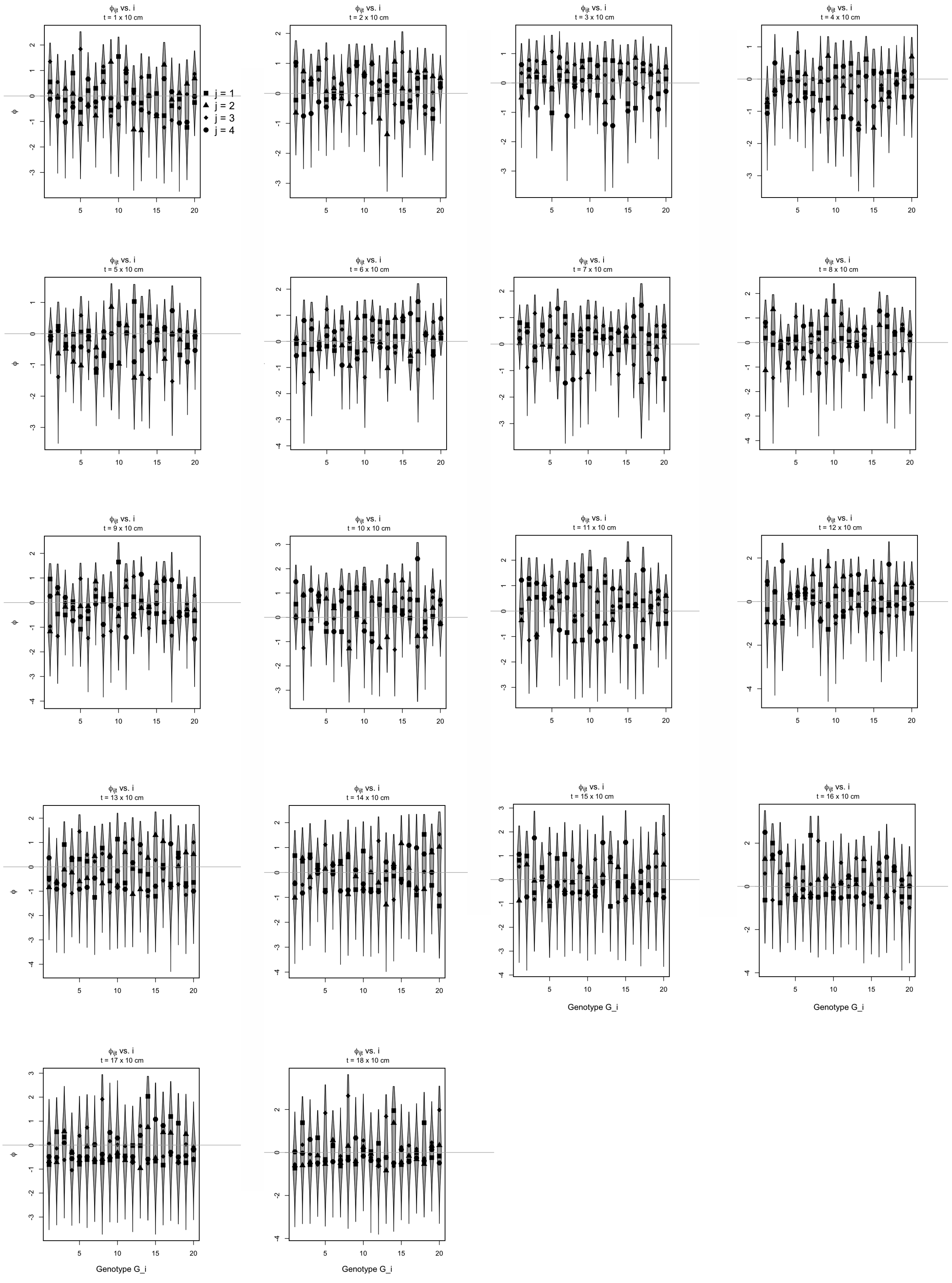

Figure S4. Violin plots: Level 1 noise ϕ against genotype i; each panel is associated with a particular depth t. Inside the tth panel are 20 genotype-specific violin plots, each $\{i, t\}$-th violin corresponding to pooling four plot-specific violins (over blocks $j = 1, 2, 3, 4$). The plot-specific medians of ϕ are shown in black (see top-left panel for legend). Minor anomalies are apparent at the highest values of t.

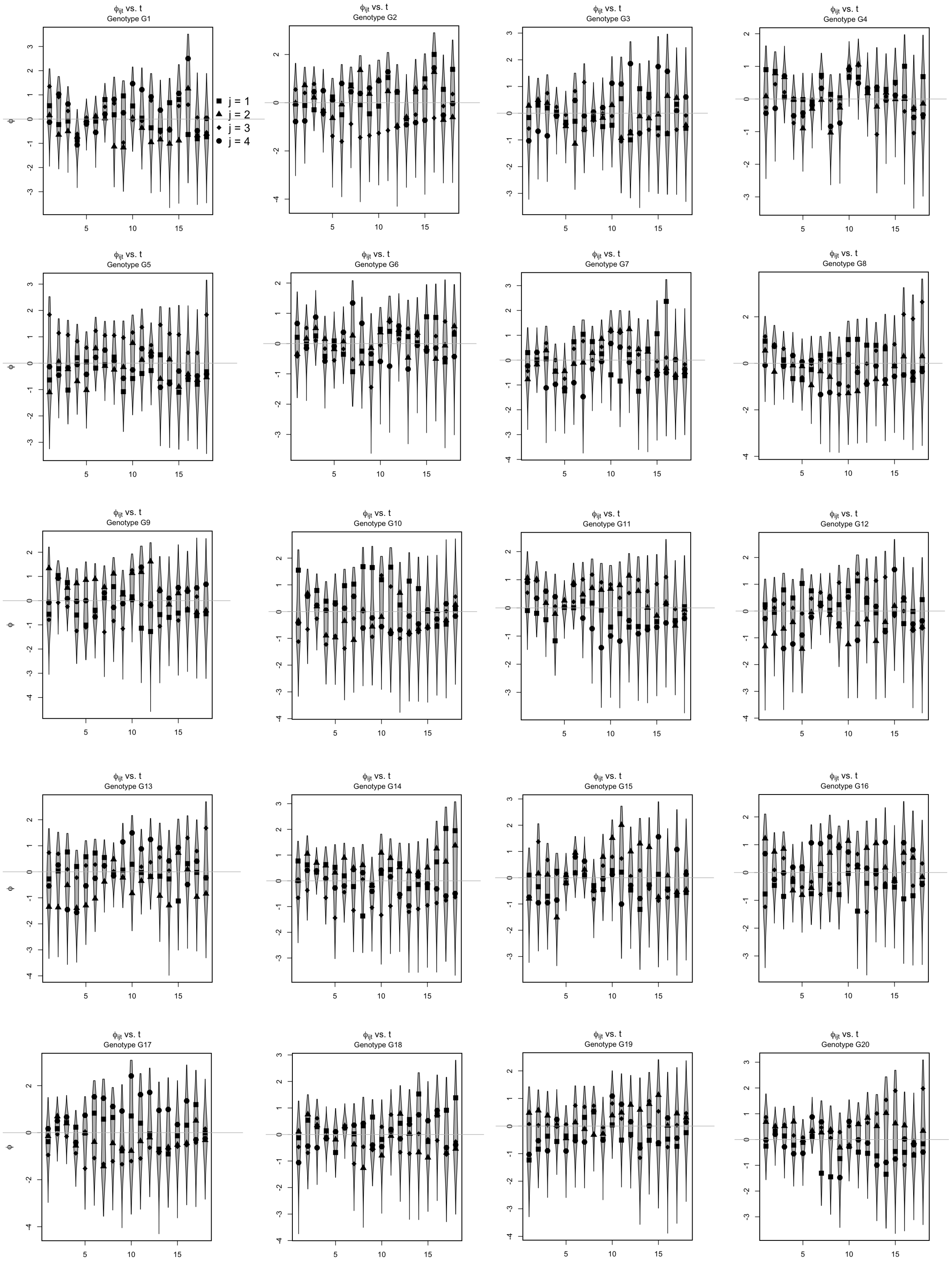

Figure S5. Same as Figure S4, except for the role reversal between genotype i and depth index t. Minor anomalies over higher values of t are apparent at isolated values of i and/or j.

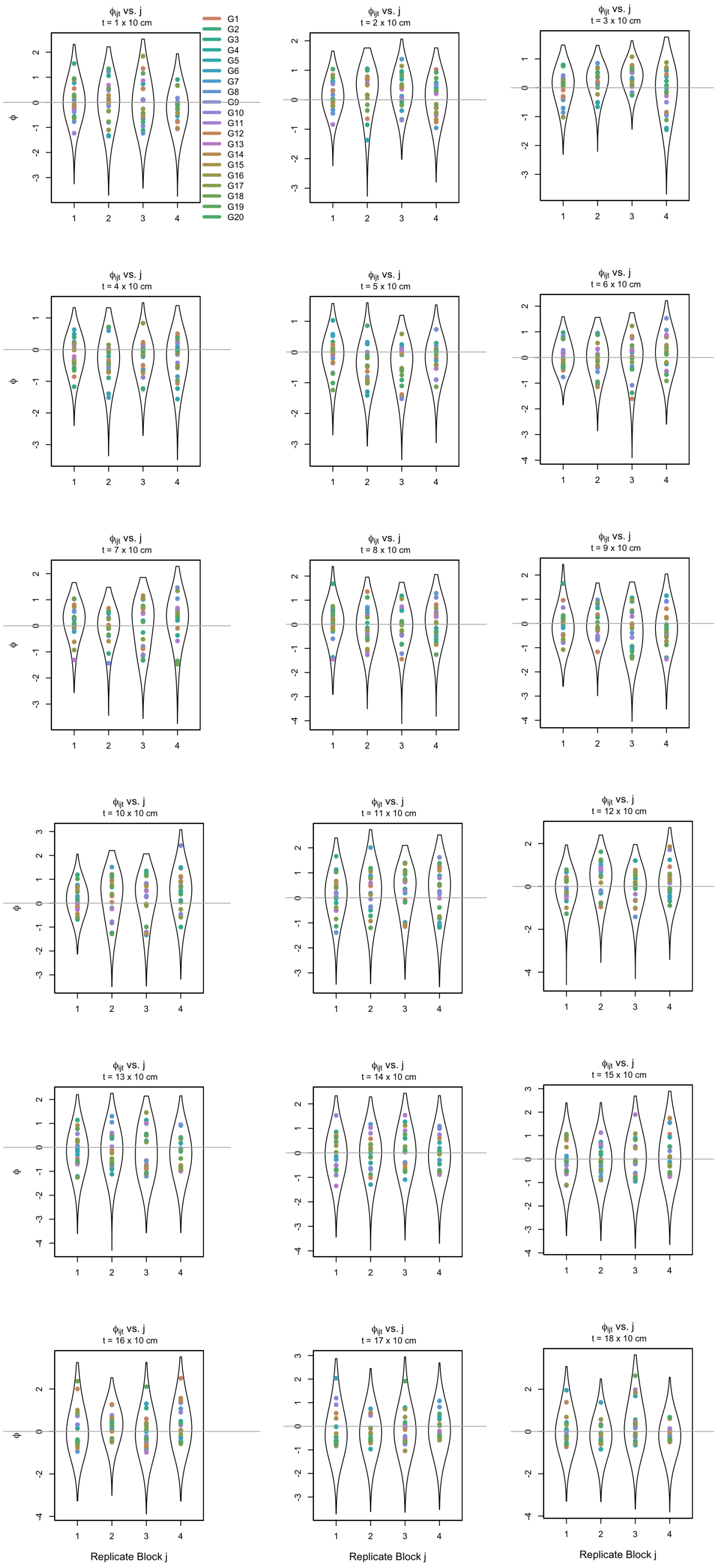

Figure S6. Violin plots: Level 1 noise ϕ against replicate block j; each panel is associated with a particular depth t. Inside the tth panel are 4 block-specific violin plots, each $\{j, t\}$-th violin corresponding to pooling 20 genotype-specific violins (over genotypes $i = G1, ..., G20$). The genotype-specific medians of ϕ are shown in color (see top-left panel for legend). Minor anomalies are apparent at various values of t.
